# RETRACTED: Wang et al. Nanostructured Nickel Nitride with Reduced Graphene Oxide Composite Bifunctional Electrocatalysts for an Efficient Water-Urea Splitting. *Nanomaterials* 2019, *9*, 1583

**DOI:** 10.3390/nano15070491

**Published:** 2025-03-26

**Authors:** Feng Wang, Dongsheng Zhao, Linbao Zhang, Liming Fan, Xiutang Zhang, Shengnan Hu

**Affiliations:** 1 Department of Chemistry, College of Science, North University of China, Taiyuan 030051, China; w1834251187@163.com (F.W.); zd484669106@163.com (D.Z.); 2 State Key Laboratory Base of Eco-Chemical Engineering, College of Chemistry and Molecular Engineering, Qingdao University of Science & Technology, Qingdao 266042, China; zhang_linbao@126.com; 3 Wuhan Hudiandian Technology Co., Ltd., Wuhan 430000, China

The journal retracts the article titled “Nanostructured Nickel Nitride with Reduced Graphene Oxide Composite Bifunctional Electrocatalysts for an Efficient Water-Urea Splitting” [1], cited above.

Following the publication, concerns were brought to the attention of the Editorial Office regarding the scientific accuracy of the data presented in the published paper [1].

Accordingly, an investigation was conducted by the Editorial Office and Editorial Board, which confirmed inconsistencies within the XRD data presented in Figures 1, 2 and 5. Following discussions, the Editorial Board and the authors have decided to retract this article [1], as per MDPI’s retraction policy (https://www.mdpi.com/ethics#_bookmark30, accessed on 15 October 2024).

This retraction was approved by the Editor-in-Chief of the *Nanomaterials.*

Feng Wang, Dongsheng Zhao, Linbao Zhang, Liming Fan and Shengnan Hu agreed to this retraction. Xiutang Zhang did not provide a comment on this decision.
